# AGE AS ONE OF THE DETERMINANTS OF RESILIENCE TO THE COVID-19 PANDEMIC AND ITS IMPACTS ON SEX LIFE

**DOI:** 10.1093/geroni/igac059.795

**Published:** 2022-12-20

**Authors:** Liza Berdychevsky

**Affiliations:** University of Illinois at Urbana-Champaign, Champaign, Illinois, United States

## Abstract

Recent research started exploring the impacts of the COVID-19 pandemic on sex life. However, there is a dearth of research on the roles of age in the overall adjustment to the pandemic and its impacts on sex life. The purpose of this study was to address this gap. Data collection was based on the online survey methodology (N=675, age range:18-76yo). Data analysis included one-way ANOVA and multivariate multiple regression. Age differences were found in the participants’ overall ability to adjust to the pandemic-related lockdown conditions, with older adults (51+yo group) adjusting better than the rest of the sample and young adults (18-30yo group) adjusting significantly worse. Resilience to the impacts of COVID-19 on sex life also varied by age. Sexual desire deteriorated the worst among middle-aged participants (31-50yo group); likely, due to increased childcare responsibilities. However, the issues with physical sexual difficulties have worsened the most among older adults. Regression results also showed that age had negative effects on the frequencies of intimate and sexual behaviors (sexual touching and caressing, sexual intercourse, and oral sex) and sexual desire and satisfaction with sex life during the pandemic. While older adults’ overall adjustment capacity to the pandemic’s conditions was the highest in the sample, the pandemic’s impacts on their sex lives and sexual health were the worst. The pandemic has exacerbated existing vulnerabilities and further jeopardized older adults’ sexual wellbeing. This study provides age-specific insights for sexual counseling and age-targeted sexual health education for older adults during the pandemic and its aftermath.

